# Nanomedicine marvels: crafting the future of cancer therapy with innovative statin nano-formulation strategies

**DOI:** 10.1039/d4na00808a

**Published:** 2024-10-18

**Authors:** Ashkan Karimi Jirandehi, Reza Asgari, Sanaz Keshavarz Shahbaz, Nima Rezaei

**Affiliations:** a Student Research Committee, School of Medicine, Qazvin University of Medical Sciences Qazvin Iran; b USERN Office, Qazvin University of Medical Science Qazvin Iran; c Cellular and Molecular Research Center, Research Institute for Prevention of Noncommunicable Disease, Qazvin University of Medical Sciences Qazvin Iran Skeshavarzshahbaz@gmail.com; d Research Center for Immunodeficiencies, Pediatrics Center of Excellence, Children's Medical Center, Tehran University of Medical Science Tehran Iran Rezaei_nima@tums.ac.ir; e Primary Immunodeficiency Diseases Network (PIDNet), Universal Scientific Education and Research Network (USERN) Tehran Iran

## Abstract

Statins, traditionally used for managing hyperlipidemia and cardiovascular diseases, have garnered significant interest for their potential anti-cancer properties. Research indicates that statins can inhibit critical processes in cancer development, such as apoptosis, angiogenesis, and metastasis. Despite their promising anti-cancer effects, the clinical application of statins in oncology has been hampered by their inherent low solubility and bioavailability. These pharmacokinetic challenges can be effectively addressed through the use of nano-based drug delivery systems. Nano-formulations enhance the delivery and therapeutic efficacy of statins by improving their solubility, stability, and targeting ability, thus maximizing their concentration within the tumor microenvironment and minimizing systemic side effects. This review delves into the potential of nanoparticles as carriers for statins in cancer therapy. It explores the mechanisms by which statins exert their anti-cancer effects, such as through the inhibition of the mevalonate pathway, modulation of immune responses, and induction of apoptosis. Furthermore, the review examines the development of various statin-loaded nano-formulations, highlighting their advantages over conventional formulations. The novelty of this review lies in its focus on recent advancements in nanoformulations that enhance statin delivery to the tumor microenvironment. By discussing the current advancements and prospects of statin nano-formulations, this review aims to provide a comprehensive understanding of how these innovative strategies can improve cancer treatment outcomes and enhance the quality of life for patients. The integration of nanotechnology with statin therapy offers a novel approach to overcoming existing therapeutic limitations and paving the way for more effective and safer cancer treatments.

## Abstract 2

The use of statins in cancer therapy is promising. Nanoformulations of statins offer higher bioavailability, increased tumor concentration, better drug resistance reversal, and fewer side effects compared to free forms.

## Graphical abstract

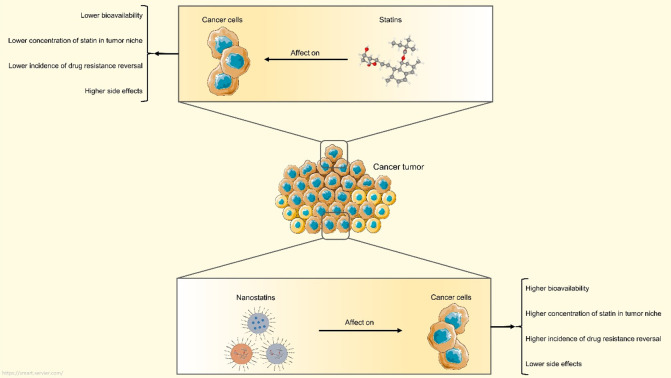

## 1. Introduction

Cancer, which is known as an abnormal division of cells without any control with the chance of invasion into other tissues, is one of the significant global diseases. According to estimations, there were 20 million new cases of cancer and 10 million cancer-related deaths globally in 2022, which highlights the huge burden of this disease.^1,2^ Although cancer is a non-communicable disease, the prevalence and the new cases of cancer incidence continue to rise annually. For example, in 2023, an estimated 1 958 310 new cancer cases were detected in the United States, with cancer being the leading cause of death after heart disease, accounting for approximately 605 000 deaths. The situation was worse during the COVID-19 pandemic as most of the attention was focused on managing COVID-19 patients, leading to delays in cancer diagnosis and treatment.^2^

Cancer is mostly a multifactorial disease like diabetes. Most of the critical risk factors are related to the environment and lifestyle. On the other hand, most of the cancer treatments are invasive and have a lot of side effects. Therefore, prevention of risk factors and lifestyle modification is currently the safest way to manage cancer. However, less invasive and safer treatments will likely be developed in the foreseeable future.^3,4^

There are a lot of different treatments for cancer, and the cancer stage is one of the most important factors in treatment effectiveness. The available cancer treatments include surgery and chemotherapy, which are the oldest and the most well-known. Other treatments include radiation therapy, hyperthermia, immunotherapy, stem cell transplant, and targeted therapy. The main limitation of cancer treatments is their side effects and invasiveness. New therapies such as targeted therapy or nano-formulation chemotherapy are more precise and have limited side effects.^1,5–7^

Among the various cancer treatments, chemotherapy is notably less invasive compared to others. Utilizing drugs that offer high efficacy with minimal side effects in nano-formulations could be an ideal approach to cancer therapy. Using nanotechnology in drug delivery, modern vaccines, diagnostics, *etc.* is a new aspect of the treatment of cancer nowadays.^8^ Also, there is a high diversity in applicable methods and materials for cancer therapy especially for drug delivery, such as polymeric or non-polymeric nanomedicine, lipophilic, hydrophilic, and metallic nanoparticles, and other similar things.^9,10^ The diversity and efficacy of nanotech drug delivery make it a high potential treatment for the future of cancer therapy. The statin drug family, known for its anti-atherosclerotic effects, has shown promising anti-cancer potential. Statins are widely recognized for their role in lowering cholesterol and treating cardiovascular diseases. They function primarily by inhibiting 3-hydroxy-3-methyl-glutaryl-CoA reductase (HMGCR), a key enzyme in cholesterol biosynthesis. Despite their therapeutic benefits, statins face limitations due to their low solubility and bioavailability, which restrict their clinical application as anti-cancer agents.^5,11^

Recent developments in nanotechnology offer promising solutions to overcome the limitations of statins in cancer treatment. Nano-based drug delivery systems enhance the solubility, stability, and bioavailability of statins, improving their therapeutic efficacy and reducing systemic side effects. Given the potential of statins as anti-cancer agents and the advantages of nanotechnology, this review aims to explore the role of statin-loaded nano-formulations in cancer therapy. The primary objective of this review is to provide a comprehensive analysis of recent advancements in the development and preclinical evaluation of statin-loaded nanoparticles and to assess their potential to enhance cancer treatment outcomes. This review also seeks to highlight the underlying mechanisms by which statins exert anti-cancer effects when delivered *via* nanoformulations, offering insights into future research and clinical applications.

## 2. Statins

In order to treat elevated cholesterol and atherosclerosis, statins have been approved.^12^ In addition to lowering lipid levels, statins are important inhibitors of 3-hydroxy-3-methyl-glutaryl-CoA reductase (HMGCR). The effect of statins on plasma cholesterol levels results from a lowering in *de novo* cholesterol biosynthesis, as well as modifications in the expression of LDL receptors.^13^ Despite differences in their side chain structures, all-natural statins possess a polyketide ring structure, the hydroxy-hexahydro naphthalene ring system, that is attached at C8 and C6 to the ring.^14^ Therefore, they are classified into different types based on their chemical structure and formation.

### 2.1. Types

In the current clinical trial, five statins are being used, which can be classified as natural, semi-synthetic, or synthetic, based on their origin.

Simvastatin, unlike natural statins (lovastatin and pravastatin), is a semi-synthetic statin derived from lovastatin. Mevalonate and pyridine, respectively, are the sources of atorvastatin and fluvastatin, two fully synthetic statins.^15^ It is believed that natural statins (lovastatin and pravastatin) are secondary metabolites of fungi which can be produced by various filamentous fungi such as Aspergillus terreus or *Monascus* ruber. Aspergillus terreus or *Monascus* ruber are two organisms that can ferment lovastatin. On the other hand, as a result of a chemical transformation that occurs with mevastatin (a second metabolite produced by *Penicillium citrinum*), pravastatin is obtained. The chemical modification of lovastatin's side chain at position C8 results in simvastatin, a semi-synthetic derivative of lovastatin.^14^ Another type of division can also be mentioned: different statins have varying solubility profiles, which necessitates distinct formulation strategies to optimize their delivery and therapeutic efficacy in cancer treatment. For example, pravastatin and rosuvastatin are hydrophilic, while simvastatin and atorvastatin are lipophilic. Hydrophilic statins like pravastatin and rosuvastatin may benefit from techniques to help them cross cell membranes and reach intracellular targets. Conversely, lipophilic statins like simvastatin and atorvastatin may require nanoformulations to enhance their solubility and stability in the bloodstream. To maximize statins' anti-cancer potential, nanoformulation strategies must be customized to their specific physicochemical properties.^16^ Therefore, they can be effective in different ways depending on their different structures, as we will discuss further.

### 2.2. Mechanism

Recent research revealed that statins decrease cholesterol synthesis, mainly in the liver, where they are selectively distributed and modulate lipid metabolism, by inhibiting HMG-CoA reductase. The percentage decrease in LDL cholesterol is positively correlated with statin antiatherosclerotic effects. Moreover, they can suppress atherosclerosis independently of their hypolipidemic effect.^17^ The molecular mechanisms underlying statin immunomodulation often involve multiple pathways, as they regulate genes encoding key molecules involved in antigen presentation and immunomodulation. In addition, it is reported that nuclear factor kappa-B, which is responsible for the transcription of numerous immunologic genes, is downregulated by statins.^18^

Furthermore, statins may suppress cancer growth through their anti-angiogenic, immunomodulatory, and pro-apoptotic effects. Statins have been shown to inhibit a variety of cancer cells, although the sensitivity to statin-induced cell death varies between different types of cancer cells. Many types of cancer cell lines are affected by their pro-apoptotic effects. It has been suggested that statins have multiple anti-angiogenic effects by inhibiting angiogenesis through the downregulation of pro-angiogenic factors and inhibiting endothelial cell proliferation and extracellular adhesion by blocking intercellular adhesion of molecules.^18^ Different mechanisms of statins are likely to have different clinical applications in different parts of the body, as we will discuss in more detail.

### 2.3. Clinical application

Hyperlipidemia is characterized by high levels of blood triglycerides and cholesterol, so statins are commonly used to treat the condition.^19^ Individuals with an elevated risk of developing cardiovascular disease (CVD) often utilize statins to decrease their risk of experiencing cardiovascular events such as heart attacks, strokes, and other related occurrences. The administration of statins has been demonstrated to significantly decrease the likelihood of such events.^20^ Individuals with a history of stroke or at a high risk for stroke may benefit from statins as they can reduce the stroke risk.^21^ Furthermore, statins are used to prevent peripheral artery disease, a condition affecting the blood vessels in the legs and arms.^22^ Familial hypercholesterolemia, a genetic condition that results in high blood cholesterol levels, can also be treated with statins. Due to their anti-inflammatory properties, statins have proven to be effective in treating certain conditions, such as rheumatoid arthritis.^23^ Evidence indicated that statins could be effective in preventing dementia in individuals who are at high risk for the condition.^24^ Researchers have shown that statins can improve liver function and reduce inflammation in patients with non-alcoholic fatty liver disease (NAFLD).^25^ Chronic kidney disease patients take statins to reduce their risk of cardiovascular events and slow the progression of the disease.^26^ Additionally, research suggests that statins can be effective in the prevention of venous thromboembolism, a medical condition marked by the formation of blood clots in the veins.^27^

Some studies suggest that statins may prevent breast, prostate, and colorectal cancer in some people. Statin users have a lower risk of death from prostate cancer, according to data from prospective observational studies. Statins are indirectly associated with advanced and fatal prostate cancer through serum cholesterol reduction.^28^ In recent years, several scholars have suggested that simvastatin may be an effective treatment for colorectal cancer, and numerous clinical studies have confirmed that simvastatin inhibits endothelial cell proliferation and migration, resulting in apoptosis, which has clinical anticancer effects.^29,30^ On the other hand, statins reduce breast cancer by increasing apoptosis and radiosensitivity in breast cancer cells, inhibiting the proliferation, and invasion of breast cancer cells. Furthermore, recent research indicated that statins can improve local control, decrease metastatic dissemination of tumors, and reduce mortality in clinical trials of statin users. Inflammatory and triple-negative breast cancers, which commonly have poor outcomes, may benefit from statins, according to recent research. Invasive breast disease may be promoted by statins over time, so caution should be exercised when prescribing them. Further research is needed to determine the type, duration, and timing of statin administration.^31^ The use of nano-based drug delivery systems for statins is justified for cancer treatment, given the significant challenges posed by the low solubility and bioavailability of statins when administered orally. Nanoformulations improve statin delivery, solubility, stability, and targeted tumor delivery for better therapeutic outcomes and patient quality of life.^32^ As mentioned in this section, one of the most important uses of statins is their anti-tumor effect, which we will discuss in more detail later.

## 3. Anti-tumor effects of statins

Statins are widely known as lipid-modulator drugs that reduce cholesterol levels through the inhibition of 3-hydroxy-3-methyl-glutaryl-CoA (HMG-CoA) reductase. However, studies have shown that statins have pleiotropic effects, including effects on diabetes, neurological disease, inflammation, and cancer, independent of their lipid-lowering effect.^11,33^

The cytotoxic potential and anti-cancer properties of statins have attracted a lot of attention. Statins, especially lipophilic statins such as simvastatin, have different kinds of mechanisms for destroying cancer cells, including the pro-apoptotic effect, inhibition of tumor cell growth, inhibition of the mevalonate pathway, and so on. In the following, the important anti-tumor effects of statins have been discussed.^11,14^

### 3.1. Inhibition of tumor cell growth

Cell growth has been boosted in the tumor cell and because of this situation, tumor cells proliferate rapidly, and uncontrolled tumor cell proliferation can lead to tumor metastasis.^33^ Statins inhibit tumor cell growth through different mechanisms that decrease both cell survival and proliferation.^34^ Thus, one of the important mechanisms is inducing apoptosis which will be thoroughly discussed in another section. In this part, other critical pathways will be reviewed.

**Fig. 1:**
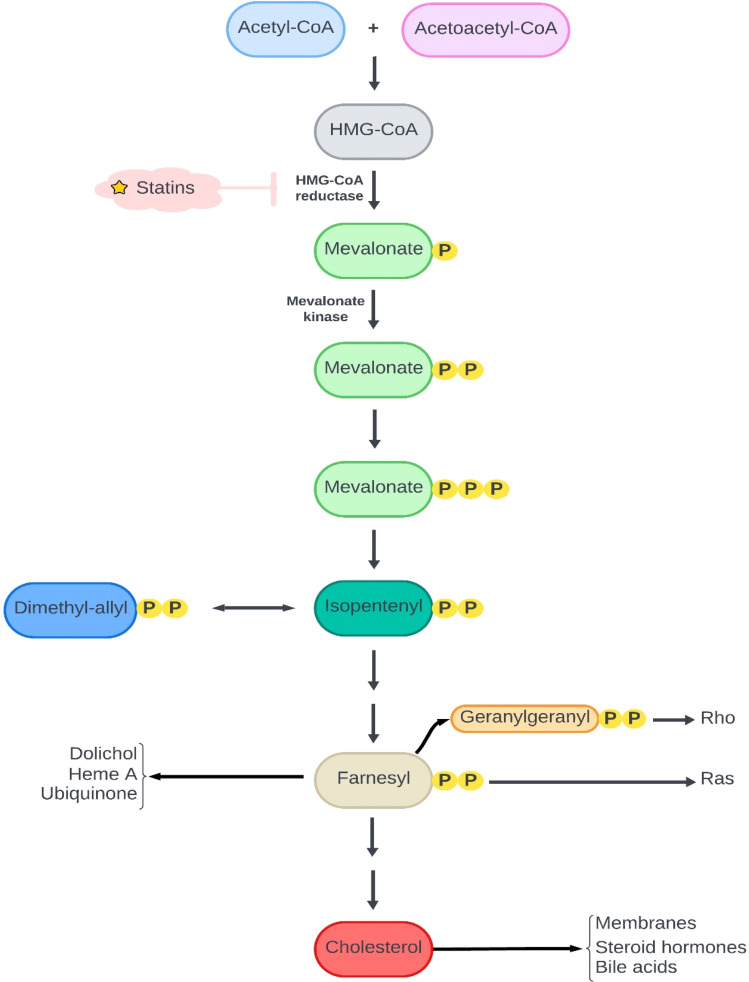
The mevalonate pathway, one of the best-known pathways in the mechanism of inhibition of tumor cell growth. There are a lot of critical mediators in this pathway for cell survival and division. The need for these mediators increases in cancer cells and accordingly, statins can restrict the cell's survival and division *via* the reduction in the production of critical mediators of the mevalonate pathway.

Changing the normal metabolism is a hallmark of cancer cells.^35^ Uncontrolled proliferation and growth lead to this alteration in the metabolism. The mevalonate pathway is one of the vital pathways in cell metabolism, producing various isoprenoids necessary for all cells, including cholesterol, polyols, ubiquinone, vitamin D, and lipoproteins.^33,35^ Several studies have shown increased mevalonate pathway demand in oncogenic situations, making suppression of this pathway a potential cancer therapy (Fig. 1). Statins inhibit the mevalonate pathway by inhibiting HMG-CoA reductase. Statin-mediated inhibition of the mevalonate pathway leads to the inhibition of tumor cell growth, proliferation, and metastasis.^33,35,36^ One study showed that the administration of exogenous mevalonate reversed the effects of statins on cancer cells.^36^

The hippo pathway, discovered in 2003 as a controller signaling for cell proliferation and apoptosis, cross-talks with the mevalonate pathway. Transcriptional co-activator with a PDZ-binding motif (TAZ) has oncogenic roles and is placed downstream of the hippo pathway.^37,38^ TAZ is significantly expressed in some cancers, such as hepatocellular carcinoma (HCC). Administration of statins in HCC leads to the inhibition of tumor cell proliferation through the inhibition of TAZ expression. Statins may indirectly inhibit TAZ expression by decreasing the expression of the TAZ upstream (LATS1, LATS2, and microRNA-9-3p). Statins may affect the hippo pathway and TAZ expression *via* the mevalonate pathway.^39^

Upregulation of the Ras family is common in cancers, such as small-cell lung cancer.^40^ Ras family proteins are upstream of two major intracellular signaling pathways, mitogen-activated Erk kinase (MEK)/extracellular signal-regulated kinase (Erk) and phosphoinositide 3-kinase (PI3K)/protein kinase B (PKB). MEK/Erk and PI3K/PKB pathways respond to polypeptide growth factors and play an important role in cell growth and proliferation.^17,40^ The use of statins inhibits prenylation of the Ras superfamily, causing anti-proliferation and anti-metastatic effects by blocking several pathways such as MEK/Erk and PI3K/PKB.^40,41^

PI3K activates PKB/AKT, then AKT or PKB activates mTOR downstream. After activation of some other intermediate messenger, gene expression changes. AKT, upstream of mTOR, is known as a critical factor for the survival, motility, and proliferation of cells.^42^

AKT/PKB activates NFκB in another way. NFκB activation represses PTEN expression. PTEN is a tumor suppressor with anti-proliferation and pro-apoptotic effects. Statins' inhibition of the PI3K/AKT pathway results in NFκB inhibition, which leads to depression of PTEN. PTEN activation causes anti-proliferation effects.^43^

Studies have shown that IL-6 can significantly increase proliferation and metastasis in cancers, such as renal cell carcinoma (RCC) due to the activation of JAK2/STAT3, a pro-inflammatory pathway. Statins can neutralize the effects of the IL-6-induced JAK2/STAT3 pathway.^42,44,45^

Some studies have focused on cell cycle-regulating proteins to define the anti-tumor effects of statins. Statins can arrest the cell cycle in the G0/G1 phase. Investigations have shown that cyclin-dependent kinases, such as CDK1, CDK2, CDK4, and cyclins D1 and E, are downregulated, while cyclin-dependent kinase inhibitors, such as p19 and p27, are upregulated when statins are taken.^46^

### 3.2. Inducing apoptosis

Programmed cell death or apoptosis is another way to stop uncontrollable cell proliferation or growth. Unlike other cell deaths, apoptosis produces no damage-associated molecular pattern (DAMP) or inflammatory cytokine.^47^ Activation of apoptosis has two pathways, intrinsic and extrinsic.^48^ Due to the activation of apoptosis through both intrinsic and extrinsic pathways, statins can be one of the most suitable drugs for chemotherapy.^49,50^

There are some intermediate products in the mevalonate pathway which are related to the activation of apoptosis. An experiment has shown that adding some of the mevalonate pathway substances, such as mevalonate, geranylgeranyl pyrophosphate (GGPP), and farnesyl pyrophosphate (FPP), inhibits cell apoptosis. The statin apoptosis activation mainly refers to the lack of GGPP. Lack of GGPP results in the reduction of protein geranylgeranylation of the Rho family.^49,51^

Statins increase the expression of Bax, the pro-apoptotic factor, and decrease the expression of BCL-2, the anti-apoptotic factor, in the intrinsic or mitochondrial pathway of apoptosis. Naturally, BCL-2 decreases the chance of apoptosis by inhibiting the Bax function. Cancer cells inhibit apoptosis to increase cell surveillance, but statins increase the cytoplasm concentration of the cytochrome C *via* increasing the penetration of the mitochondrial membrane through upregulation of Bax in the mitochondrial membrane. Therefore, cytochrome C triggers the apoptosis cascade. In general, imbalanced anti- and pro-apoptotic protein expression, in favor of pro-apoptotic proteins, leads to apoptosis.^52,53^

As discussed in the last part, some pathways are important for cell survival, such as PI3K/AKT, MEK/Erk, and c-Jun-N-terminal kinase (JNK) pathways, and so on. These pathways maintain cell survival through the inhibition of the apoptosis cascade directly or indirectly. Therefore, statins can facilitate the activation of apoptosis by inhibiting these pathways.^53,54^

Statins cause a disturbance in lipid rafts. On the other hand, statins upregulate Fas-ligands (FasL) on the surface of cancer cells. All these changes lead to ligand-independent FasL clustering. FasL clusterization results in unnecessary activation of caspase-8 and apoptosis in the extrinsic pathway.^11,50,55^

Besides apoptotic effects, statins also can induce other kinds of cell death such as autophagic cell death, ferroptosis, and pyroptosis.^33^

### 3.3. Inhibition of angiogenesis

According to experimental studies, statins protect the heart against ischemia-reperfusion injury and stimulate the growth of new blood vessels in normocholesterolemic animals with ischemic limbs. However, higher doses of statins can also inhibit angiogenesis and endothelial cell migration, which contradicts these findings. While the mechanisms behind statin effects on endothelial function are not completely understood, there is increasing evidence that statins influence endothelial function *via* endothelial-derived nitric oxide. It has been discovered that statins regulate multiple angiogenic processes in endothelial cells by regulating the serine/threonine protein kinase Akt.^56,57^ In particular, it is important to understand how statins affect angiogenic mediators and how their effects differ at low and high doses.

When statins are administered at low concentrations (nM range), which bears close resemblance to long-term statin therapy, plasma concentrations and phosphatidylinositol-3-kinase-protein kinase B (PI3K-Akt) pathways (an intracellular signal transduction pathway) promote angiogenesis and result in the release of nitric oxide. Angiogenesis is stimulated by nitric oxide, which stimulates cell proliferation, migration, and differentiation in the endothelium, thus promoting angiogenesis.^58,59^

In contrast, apoptosis-mediated antiangiogenic effects result from statins at high concentrations (M range or higher).^58^ Statins have been shown to prevent angiogenic growth by decreasing vascular endothelial growth factor (VEGF) release in endothelial cells and increasing endothelial apoptosis;^57,60^ this may occur through reducing geranylgeranylation of Rho proteins, which modulate vascular endothelial growth factor receptor-2 (VEGFR-2) activity.^61^ Among the antiangiogenic effects of statins are inhibiting the activity of monocyte chemoattractant protein-1,^62^ metalloproteinase, and angiotensin-2,^63,64^ the pre-proendothelin gene, as well as actin filament and focal adhesion formation.^61^ Also, it has been shown that statins reduce T-lymphocyte activation and cytokine activity, thereby interfering with the proangiogenic effect affected by inflammation.^61^ Aside from its inhibitory effects on angiogenesis, statins may also have an inhibitory effect on the invasion and metastatic spread of various tumors.

### 3.4. Inhibition of tumor invasion and metastasis

Currently, statins are used widely in clinical practice; therefore, they may have future clinical applications if found to inhibit tumor metastasis.

According to experimental data, a significant reduction in lung metastasis, cell migration, invasion, and adhesion was observed with statins at concentrations that did not have a cytotoxic effect on B16BL6 cells.^65^ Recent evidence has shown that statins inhibit the growth of tumors, invasion, and metastasis formation in cancer cells. Due to their ability to block isoprenoid production, statins interfere with post-translational modifications that occur in a wide variety of proteins, including those involved in normal cell signaling. This suggests that statins may protect against some types of cancer.^66,67^ Accordingly, statins may inhibit tumor growth and metastasis. Furthermore, another study examined the relationship between statins, cancer metastasis, and autophagy. In this study, statins promote autophagy in cancer cells, which helps prevent metastatic spread. Adenosine monophosphate-activated protein kinase-target of rapamycin (AMPK-TOR) signaling is a key mechanism that statins promote autophagy through. Indeed, it is obvious that autophagy has both anti-metastasis and pro-metastasis properties in tumor metastasis.^68^ In addition to their effectiveness in treating colorectal cancer, statins may also reduce its invasive and metastatic potential. This hypothesis, however, is supported only by limited data, and further studies are needed to assess the validity of this hypothesis.^69^*In vitro* and *in vivo* studies have found that simvastatin inhibits the ability of triple-negative breast cancer (TNBC) cells to metastasize.^70^ Researchers found that simvastatin, a lipophilic statin, can inhibit cancer-initiating cells' metastatic potential. The expression pattern of stemness and epithelial-mesenchymal cell markers is modified by simvastatin, which adversely affects cancer cell assembly and spheroid formation. A 3D-mesomimetic model subsequently revealed that cancer-initiating cells had less metastatic potential and showed lower ascites/tumor burden.^71^ However, all these effects were examined separately for each statin and are dependent on their individual characteristics, as we will discuss later.

### 3.5. Reversion of multidrug resistance

Some investigations use common anti-cancer drugs in combination with statins. For example, a study investigated the effect of simvastatin and capecitabine combination on gastric cancer.^72^ In another study, in which a lipid nanoemulsion was used for delivering the drug, the effect of combined simvastatin-doxorubicin was investigated on MCF-7 breast cancer and HFS Human Foreskin Cells.^73^ Another study was carried out to discover a new treatment for glioblastoma.^74^ In this study, the combination of pitavastatin and irinotecan, a topoisomerase 1 inhibitor for cancer treatment, was investigated.

All the mentioned studies showed that the combination of statin and the anti-cancer drug has a kind of synergic effect in the treatment of cancer compared to the use of anti-cancer drugs alone. Also in further studies, scientists showed that using statins in the chemotherapies can prevent or reverse the multidrug resistance phenomenon.

Studies propose that statins can decrease the activation of NF-κB *via* suppression of RhoA GTPase activity. When the activation of NF-κB has been decreased, it can lead to a decrease in the expression of the genes that are related to the NF-κB pathway, such as cyclin D1, COX-2, Bcl, survivin, and MMP9.^14^ Finally, all the changes in the gene expression disrupt the function of specific transporters such as transporters P-glycoprotein (PGP), multidrug resistance-associated protein (MRP)-3, and ATP-binding cassette (ABC).^75^ These specific transporters can transport the anti-cancer drug to the extra-cellular fluid (ECF) and prevent the accumulation of the drug inside the cancer cells. Using statins in combination with anti-cancer drugs, along with their anti-cancer effects, can inhibit these transporters and facilitate the accumulation of the anti-cancer drug in cancer cells. Thanks to this function of statins, lower dosages of anti-cancer drugs can be used, and it will cause fewer side effects for patients.^11^

**Fig. 2:**
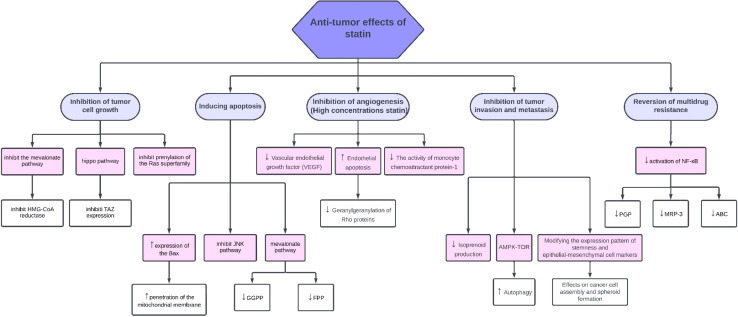
Summary of all the anti-tumor effects of statin. There are different kinds of mechanisms by which statins, especially lipophilic statins including simvastatin, destroy cancer cells, such as pro-apoptotic effects, inhibition of tumor cell growth, and inhibition of mevalonate pathways.

As reviewed in Fig. 2, statins have a lot of anti-tumor effects, but it's necessary to know that statins' anti-tumor effects can vary across a spectrum, and it depends on many factors. The two important factors are the type of statin used and the type of cancer cell being targeted. For example, lipophilic statins have higher cytotoxic effects than hydrophilic ones.^14,33^ On the other hand, some cancer cells resist statins due to their specific gene expression. These characteristics show statins' limitations which will be discussed.^36^

## 4. Limitation of statin treatment in cancer

Statins are drugs with pleiotropic effects, so they will be administered to treat many conditions such as hypercholesteremia, ventricular arrhythmias, cancer, *etc.* Despite the various benefits of statins in cancer treatment, there are some limitations in the use of statins like every other drug. The most important limitation in the administration of statins is their adverse side effects on patients. Because cancer chemotherapy is a long-term treatment, drugs will reveal their side effects more often.^53^

Myopathy is the most important and common side effect compared to the others. Also, myopathy is one of the most important indications to stop using statins. Myopathy, as a statin-associated symptom, can appear in various forms, from simple fatigue or myalgia to rhabdomyolysis which is a life-threatening condition.^53,76–78^ Statin-induced necrotizing autoimmune myopathy is another symptom that is as important and rare as rhabdomyolysis.^76,78,79^ It has been reported that diabetes mellitus and central nervous system problems are other essential side effects of statins.^78^

According to the studies, one of the reasons for the myopathy effect of statins can be statin-induced apoptosis.^53^ However, there are some other hypotheses about the mechanism of statins' side effects. For example, the negative effect of statins on selenoprotein synthesis is a hypothesis that can explain various side effects of statins. The similar appearance of selenium deficiency with statin side effects can support this hypothesis.^80^

With technological progress in the field of pharmacology, new methods and devices have been discovered or have been invented to develop the efficacy of drug effects and reduce their side effects. Nanotechnology application in drug delivery is one of the attractive proposed approaches.

## 5. Nanotechnologies' application in cancer

**Fig. 3:**
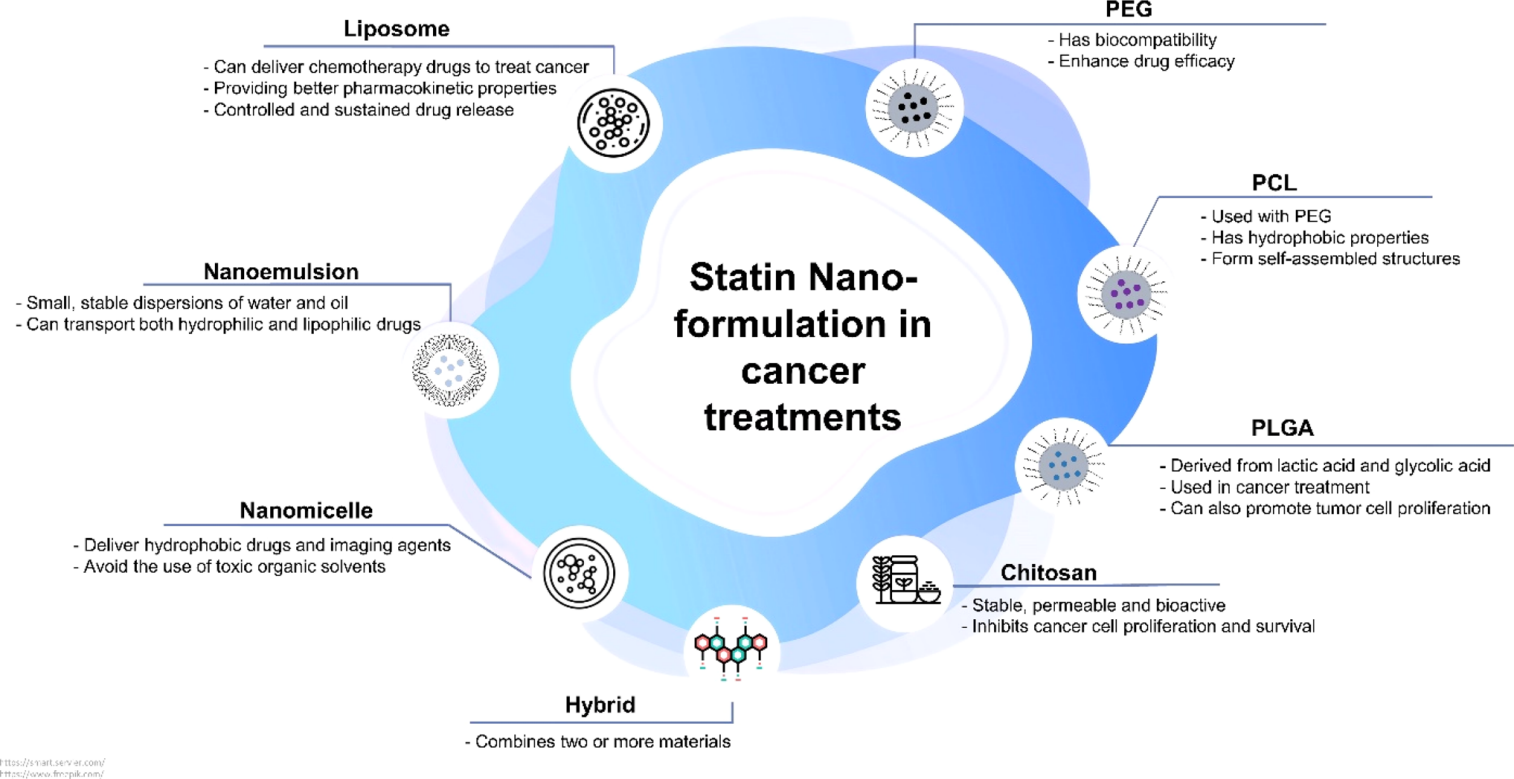
A summary of the application of statin nano-formulations in cancer treatment and their characteristics.

Recent evidence has shown that cancer treatments like chemotherapy and radiotherapy have failed, and the disease has returned. Also, in addition to causing side effects on healthy cells, cytotoxic drugs limit the amount of drug delivered to cancer cells due to their ability to affect both healthy and cancer cells.^81–83^ The primary challenges with delivering statins in cancer treatment are their low solubility and poor bioavailability, which restrict their therapeutic effectiveness when taken orally. The challenges mentioned lead to low drug concentration at the tumor site, which reduces the potential anti-cancer effects. Nanoformulations solve these issues by improving the solubility, stability, and targeted delivery of statins, ensuring higher concentrations at the tumor site and improving their therapeutic effectiveness.^84^ The use of nanoparticles as drug carriers has overcome some of the limitations of current cancer treatment methods. These structures are highly effective in delivering drugs because they protect the drug molecule, reduce toxicity and side effects, pass through biological barriers to reach the target site, and increase the drug's stability in the bloodstream, thereby enhancing the efficacy of drug therapy.^85^ As we will discuss in the next section, nanoparticles can be divided into two general categories based on whether they are polymers (like chitosan, PEG, PCL, PLGA, and their hybrids, *etc.*) or lipids (like liposomes, nanoemulsions, nanomicelles, *etc.*). Fig. 3 displays all of the mentioned categories.

Moreover, some nanomaterials have inherent therapeutic properties (for example, gold nanoshells, nanorods, and iron oxide nanoparticles) when stimulated. Cancer diagnosis and imaging can also be improved with nanoparticles. In addition, a combination of multiple drugs can be used to improve therapeutic efficacy and overcome drug resistance.^86^ Several types of cancer are treated with nanoparticles, including breast cancer, prostate cancer, brain tumors (Glioblastoma), and liver cancer.^87–90^

## 6. Statin nano-formulation in cancer treatments

Statin nano-formulations are of different types, but the lipid based and polymeric nanoparticles are the most practical and important compared to other types. The following are the most important and recent articles in this field. They are resummerized in Tables 1 and 2, respectively.

**Table 1:** Experimental and clinical outcomes of statin lipid-based nanoparticles in cancer treatments

Nanoparticle type	Statins kinds and other drugs	Cell type/animal model	Experimental outcomes	Clinical outcomes	Ref.
pH-sensitive liposomes	Co-encapsulation of Simvastatin and Doxorubicin	Human breast cancer cell lines: MDA-MB-231, MCF-7, SK-BR-3	- Better results against the MDA-MB-231 cell line by DOX and SIM at a molar ratio of 2 : 1, respectively, either in the free form or co-encapsulated into liposomes	—	94
			- The synergistic effect in treatment with SpHL-D-S (ratio of 2 : 1) with CI between 0.6 and 0.8		
			- In migration assay, significantly reduced the percentage of cell migration compared to treatments containing only DOX in all treatments containing SIM		
Liposome nanoparticle	Simvastatin	Uterine leiomyoma in a patient-derived xenograft mouse model	- Enhanced the drug delivery in simvastatin-loaded liposome	- No significant therapeutic improvement in liposomal formation of simvastatin compared to simvastatin	95
Liposome nanoparticle	Co-formulation of simvastatin and doxorubicin	C26 murine colon cancer cells co-cultured with macrophages as a simulation for a tumor niche	- Promoted release in a higher molar ratio of SIM DOX	—	96
			- More pronounced inhibitory effect in the ratio of 12 to 1 of SIM and DOX than other ratios		
Deformable liposome	Vorinostat and simvastatin codelivery	*In vitro* part: Murine Lewis lung cancer (LLC) cells, *in vivo* part: long cancer cell xenograft animal model (female C57BL/6 (18–22 g) mice)	- Increased the amounts of the anti-tumor M1 macrophages and the cytotoxic CD8+ T cells	- Changed angiogenesis pattern and revealed anti-angiogenesis features	97
			- Reduced the amounts of the pro-tumor M2 macrophages and regulatory T cells (Tregs)		
Hyaluronan-conjugated liposome	Fluvastatin	Breast cancer stem cell xenograft mouse model (BALB/C nude mice)	- HA-L-FLUVA (15 mg kg^−1^) + DOX (3 mg kg^−1^) as the best combination against breast cancer stem cells	- Mice survival increased after administration of HA-L-FLUVA (15 mg kg^−1^) + DOX (3 mg kg^−1^)	98
Liposome	Simvastatin and gefitinib	Brain metastasis of non-small cell lung cancer mouse model	- Reversed cancer resistance	- Inhibited tumor growth in the mouse brain	99
			- Increased penetration through the brain barrier		
			- Increased drug concentration in the brain metastatic tumor		
Liposome	Lovastatin and doxorubicin	H22 mice model (liver cancer model)	- Reduced pathological damage to the main tissues compared with the free drug administration	- Inhibited tumor growth in the liver cancer model	100
Herceptin-conjugated liposomes	Simvastatin and doxorubicin	*In vitro* phase: the PCa cell line PC3 and HUVEC (Human Umbilical Vein Endothelial Cells)	- Higher inhibition rates of tumor volumes in the application of Herceptin-coated liposomes compared to non-targeted liposomes	- Significant decrease in tumor growth in animal models	101
		*In vivo* phase: subcutaneous PC3 tumor-bearing Male BALB/c nude mice		- Synergistic anti-angiogenesis effect	
Liposome	Simvastatin and 5-fluorouracil	Balb/c mice C26 murine colon carcinoma model	—	- Sensitized tumor cell in both the liposomal and free administration of simvastatin and 5-fluorouracil	102
				- Reduced tumor growth in both the liposomal and free administration of simvastatin and 5-fluorouracil	
				- Revealed strongest anti-tumor effect in the liposomal form	
Hairpin-structured liposome	Simvastatin and paclitaxel	A549 and A549T non-small-cell lung cancer and PC9 cells	Administration of a nanoformulation of simvastatin in combination with paclitaxel leads to	—	103
			- Reverse epithelial-mesenchymal transition		
			- Re-polarize tumor-associated macrophages		
			- Promoting M2-to-M1 phenotype switching		
Immunoliposomes	Simvastatin	Breast cancer cells overexpressing HER2	Targeted liposomal simvastatin leads to:	—	104
			- Inhibition of the growth of target cells through inhibition of Akt and Erk pathways and induction of apoptosis		
			- High selectivity towards HER2-overexpressing breast cancer cells		
Micelle	Simvastatin and alendronate sodium	Human triple-negative breast adenocarcinoma, human prostate adenocarcinoma, and human lung adenocarcinoma	- Inhibit cell growth, and cell multiplication and result in a high percentage of late apoptotic and necrotic cells	—	111
Micellar nanoprobe	Simvastatin and photosensitizer protoporphyrin IX	C6 glioma cell line (rat), bEnd.3 cell (mouse), NIH/3 T3 (mouse), and human umbilical vein endothelial cells	- Cell cycle arrest at the G1-S phase	—	112
			- Accelerated apoptosis and necrosis		
			- Enhanced reactive oxygen species generation		
Micelle	Atorvastatin	2D culture: mouse glioma cells (CT-2A and GL261) and human glioblastoma cells U373, U251, and U87	- Inhibited cancer growth more significant compered with free atorvastatin	—	113
		3D culture: mouse glioma cells (CT-2A)	- Much higher drug concentration in the serum according to the 3D culture results		
Nanoemulsions containing multivalent intestinal transporter-targeting lipids	Atorvastatin	4T1 cell-bearing mice	- Increased bioavailability of oral administration of atorvastatin in the nanoemulsion formulation compared to free oral administration of atorvastatin	- Oral administration of ATV-loaded nanoemulsions significantly suppressed tumor growth	84
Eucalyptus oil-based nanoemulgel carrier	Levovastatin and eucalyptus oil	Tongue carcinoma HSC3 cell line	- Optimized formulation showed the best inhibitory concentration (IC50) and caspase-3 enzyme values	—	118
Lipid nanoemulsion	Doxorubicin and pravastatin	Swiss albino mice bearing Ehrlich ascites carcinoma	- No significant changes in biochemical parameters were detected compared to corresponding controls	- Significant decrease in body weight change	119
				- 217.35% increase in the mean survival time compared to the no-treatment group	
Nanoemulsion	Simvastatin and doxorubicin	Swiss albino mouse model of Ehrlich ascites carcinoma	- The less toxic effect on the hepatic tissue compared to the free drug administration	- Increase in the percentage lifespan compared to free drug administration	120
				- Reduced side effects of doxorubicin	
				- Improved the levels of all serum biochemical parameters	
Nanoemulsion	Pravastatin and doxorubicin	MCF-7 breast cancer cells and HFS Human foreskin cells	- Significantly reduced the doxorubicin side effect of human foreskin cells and red blood cells	—	73
			- The cytotoxicity against MCF-7 cells was preserved		
Solid lipid nanoparticles	Atorvastatin calcium and vinpocetine	HepG2, MCF 7 and melanoma B16 F10 cell line	- Developed and optimized a solid nanoparticle including atorvastatin calcium and vinpocetine	—	126
			- Enhanced anticancer activity of the solid nanoparticle on all three cell lines compared with free drugs		
Lipid nanoparticles	Atorvastatin	MCF-7 breast cancer cells	- The bioavailability increased in lipid nanoparticles compared with free drug	—	127
			- The suitable drug concentration for total growth inhibition and 50% growth inhibition was 27.4 μg mL^−1^ and less than 10 μg mL^−1^ respectively		

**Table 2:** Experimental and clinical outcomes of statin polymer-based NPs in cancer treatments^a^

Polymer NPs	Cell type/animal model	Experimental outcomes	Clinical outcomes	Ref.
Chitosan encapsulated simvastatin, followed by Eudragit S100 microparticles	HCT-116	- Improvement of SMV microparticles' cytotoxicity against HCT-116 colon cancer cells as compared to raw SMV	—	130
		- Enhanced accumulation of colon cancer cells in the G2/M phase		
		- Elevation in cell fraction in early, late, and total cell death		
Chitosan-coated Simvastatin	HSC-3	- Highest EE 79.67% and better stability at 4 °C	—	131
		- Enhanced biological activity of SIM, due to QRC		
		- Lower cell viability in SIM-QRC NP-loaded ISG than the other treatments		
Chitosan-TGP polyelectrolyte complex stabilized cubic nanoparticle of simvastatin	Human breast cancer (MCF-7)	- Showed high entrapment efficiency and small size	—	132
		- Exhibited better control on the growth of human breast cancer cell lines than simple simvastatin		
PR-CNG-ER	Human liver cancer (HepG2 cells)	- Good entrapment efficiency, drug loading, and sustained release over 48 h	—	133
SVCSChSNPs	- Human hepatocellular carcinoma (HepG2 cells)	- Greater inhibition of SVCSChSNPs for proliferation in HepG2 cells and high cellular uptake through ASGPR-mediated endocytosis	- Enhanced bioavailability of SMV up to 2-fold and SVA up to 1.6-fold compared to that of the pure SMV suspension	134
	- Wister albino rats			
PEG/glycerides and lipid nanocarriers loaded with simvastatin	Human breast cancer (MCF-7 cell)	- Much higher cell viability percent in SMV-loaded Labrafil® and Labrafac® NLCs compared to the drug solution	—	138
PLGA-PEG-MAN	- C57BL/6 mice hepatoma cell line Hepa1-6	- Restores the quiescence of aHSCs *via* stimulation of KLF2-NO signaling in LSECs	- Alleviates LSEC capillarization to regress the stromal microenvironment	139
	- Human hepatocellular carcinoma cell line Huh7	- Up-regulates the expression of CXCL16 in LSECs		
	- LSEC cell line SK-Hep1	- Recruits NKT cells through CXCL16 to suppress tumor progression		
	- HSC cell line LX2			
PCL–PEG–PCL and Atorvastatin and Rosuvastatin	Human breast cancer (MCF-7 cells)	- Statin-loaded nanoparticles were cytotoxic	—	140
		- Strong and dose-dependent inhibition of cell (MCF-7 line) growth by the nanoparticles compared with statins		
PCL–PEG and Simvastatin	- Breast cancer (MCF-7 cells)	- Nanoparticles showed sustained drug release kinetics	—	141
	- HFF-2	- Strong and dose-dependent inhibition of cell growth by the nanoparticles compared with statins		
SIM-PCL/PEG NFs	Gastric cancer (MKN-45 cells)	- Induce apoptosis and autophagy in 5-Fluorouracil-resistant MKN-45 (MKN-45/R) cells	—	142
		- Decrease metastasis		
Spray-dried PLGA polymeric submicron particles of simvastatin	Breast cancer (MCF-7 cell)	- Significant increase in AUC_0−24_, *T*_1/2_, and *C*_max_, and a decrease in *K*_el_, compared to those of pure SIM	—	145
		- The submicron particles acquired by nano-spray drying were found to be appropriate for the treatment of solid tumors		
GM HCl and SMV combination in dual-encapsulated PLGA	- Pancreatic cancer cell line	- Lower IC_50_ value in nanoparticles compared to the pure drug	−1.4 fold and 1.3 fold enhanced bioavailability of GM and SMV in PLGA nanoparticles compared to the drug solution	146
	- MCF-7 breast cancer cell and MIA PaCa-2 cell lines	- Higher cytotoxicity toward the cancer cells		
	- Wistar rats			
PLGA/PEI nanoparticles	- Gastric cancer (BGC-823, SGC-7901, and HGC-27 cells)	- Lower cell viability in SMV + miR-21i compared to that of free SMV	Prolong the blood circulation time of SMV compared to that of free SMV	147
	- Sprague Dawley rat	- Decreased the cell proliferation in BGC-823, SGC-7901, and HGC-27 gastric cancer cells		
		- Being significantly less cytotoxic to normal gastric mucosa cells (GES-1)		
Acid-terminated PLGA	Human prostate cancer (PC-3 cell)	- Evade the low bioavailability of SMV	—	151
		- Confer sustained release of both encapsulated and chemically conjugated SMV		
		- Enhancing the anti-cancer effect of the formula *via* magnetic targeting with the aid of the encapsulated SPIONS		
PLHNs	- Two melanoma cell lines, COLO-38 and SKMEL-28	- Increase cytotoxicity in melanoma cells for encapsulated simvastatin	Topical films were non-irritant systems and provided a sustained release kinetic profile of simvastatin	152
	- Human keratinocyte (HaCaT) and fibroblast (COS-7) cell lines	- Highlighted the key role of squalene as a nanostructuring agent of the lipid nanoparticle matrix		

a Abbreviations: HCT, human colorectal carcinoma; SMV/SIM, Simvastatin; HSC, human tongue squamous carcinoma; EE, entrapment efficacy; QRC, quercetin; NP, nanoparticle; TGP, tamarind gum polysaccharide; MCF-7, Michigan Cancer Foundation – 7; PR, pravastatin; CNG, chitosan nanogels; ER, erythrocytes; SVCSChSNPs, Simvastatin chitosan nanoparticles co-crosslinked with tripolyphosphate and chondroitin sulfate; ASGPR, asialoglycoprotein receptor; SVA, simvastatin hydroxy acid ammonium salt sodium; PEG, polyethylene glycol; PLGA, poly(lactic-*co*-glycolic acid); MAN, Mannan; NLC, nanostructured lipid carrier; aHSCs, activated hepatic stellate cells; LSECs, liver sinusoidal endothelial cells; CXCL16, chemokine C-X-C ligand 16; NKT, natural killer T; PCL, polycaprolactone; HFF, human foreskin fibroblasts; NF, nanofiber; AUC, area under the curve; *C*_max_, maximum concentration; GM, gemcitabine; HCL, hydrochloride; IC, inhibitory concentration; PEI, polyethylenimine; SPIONS, superparamagnetic iron oxide nanoparticles; PLHN, polymeric lipid hybrid nanoparticle.

### 6.1. Lipid-based nanoparticles

There are different kinds of lipid-based nanoparticles such as liposomes and micelles which have been widely used.

#### 6.1.1. Liposomes

Liposomes are known as the most practical nanoparticles among lipid-based nanoparticles. Also, compared to other kinds of nanoparticles, liposomes are most important, especially for drug delivery.

Liposomes offer several advantages, including their biocompatibility and biodegradability, as they are composed of phospholipids similar to cell membranes, making them well-tolerated and reducing toxicity risks. Their versatile drug encapsulation allows for the delivery of both hydrophilic drugs, stored in the aqueous core, and hydrophobic drugs, housed within the lipid bilayer, enabling flexible drug delivery options. Additionally, liposomes reduce toxicity by encapsulating drugs, decreasing their exposure to healthy tissues and potentially minimizing side effects, such as those caused by chemotherapeutic agents. Targeted drug delivery is another advantage, as liposomes can be functionalized with ligands like antibodies or peptides that bind specifically to cancer cell receptors, improving tumor targeting through active targeting mechanisms. Furthermore, liposomes benefit from the enhanced permeability and retention effect, which takes advantage of the leaky vasculature in tumors, allowing liposomes to accumulate in tumor tissues, making them valuable for passive targeting.^91,92^

However, there are some disadvantages to using liposomes. Stability issues, including degradation and aggregation, especially during storage, raise concerns about their shelf life in clinical applications. Without modifications such as PEGylation, liposomes have a short circulation time, as they are quickly cleared by the mononuclear phagocyte system, reducing their overall efficacy. The drug-loading capacity of liposomes can also be limited, depending on the properties of both the drug and the liposome design. Additionally, liposomes have the potential to provoke immune responses, which may lead to rapid clearance from the body or undesirable side effects.^92,93^

In cancer treatment, liposomes are particularly suitable for delivering chemotherapeutics such as doxorubicin and paclitaxel, as they can reduce toxicity while enhancing tumor-targeted drug delivery, as seen in the example of Doxil®. Liposomes are also being explored in immunotherapy for the delivery of immune-modulating agents like checkpoint inhibitors or vaccines. Moreover, liposomes are utilized in photodynamic therapy to deliver photosensitizers, which, upon activation by light, destroy cancer cells.^91^

In 2023, Jaqueline Aparecida Duarte *et al.* revealed the antitumoral effect of co-encapsulated simvastatin and doxorubicin on three different breast cancer cell lines.^94^ A novel pH-sensitive liposomal formulation had been used for the process of co-encapsulation. The effect of different ratios of doxorubicin and simvastatin had been experimented with but there was a pronounced antitumor effect at a ratio of 2 : 1 (doxorubicin : simvastatin). In another study in 2022, a uterine leiomyoma in a patient-derived xenograft mouse model was treated with simvastatin-loaded liposome.^95^ 12 mice, which had been implanted with a leiomyoma xenograft bilaterally, were divided into three groups. One group was a control, another was a simvastatin-treated group, and lastly, there was a group that was treated with simvastatin-loaded liposome. According to the results, administration of simvastatin in liposomal form couldn't provide improvement over simvastatin but the liposomal form demonstrated a suitable potential for the delivery of simvastatin. Cristina Ioana Barbălată *et al.* in 2021 studied the development of simvastatin-doxorubicin liposomes as an enhancer of the antiproliferative effect in colon cancer therapy.^96^ They found that there is a significant link between the quality attributes of liposomes and the anti-cancer features of the liposomal formulation on C26 murine colon cancer cells. In a study in 2020, it was investigated that deformable liposomal codelivery of vorinostat and simvastatin could promote anti-tumor responses through remodeling the tumor microenvironment.^97^ This study demonstrated that codelivery of vorinostat and simvastatin with deformable liposomes enhanced the intratumor infiltration ability. The drug-filled nanoparticles remodeled the tumor microenvironment with different mechanisms such as the repolarization of tumor-associated macrophages from the M2 to M1 phenotype, and anti-angiogenesis. In 2020, encapsulated fluvastatin in a hyaluronan-conjugated liposome had been repurposed as an anticancer agent against breast cancer stem cells.^98^ Experiments in this study revealed that encapsulated fluvastatin significantly inhibited the growth of breast cancer stem cells in comparison with free fluvastatin. In 2020, Weimin Yin and his colleagues investigated the efficacy of liposome codelivery for blood–brain barrier penetration and treatment of brain metastasis of non-small cell lung cancer.^99^ The results showed that administration of the simvastatin and gefitinib in liposomal form increased the penetration and concentration of the drug in the tumor microenvironment. On the other hand, administration of simvastatin reversed the drug resistance through the mechanism of elevated ROS and the suppression of the EGFR/Akt/Erk signaling pathway. Three studies in 2019 used a liposome nano-formulation as a promising and suitable drug carrier. Tianying Wang and her colleagues investigated the synergistic effect of doxorubicin and lovastatin that were co-delivered by liposomes for the therapy of liver cancer.^100^ On the other hand, Ning Li *et al.* used doxorubicin and simvastatin co-delivered with Herceptin-conjugated liposomes for prostate cancer therapy.^101^ Later, Lavinia Luput *et al.* investigated the sensitization effect of liposomal simvastatin on C26 murine colon carcinoma.^102^ In the latter study, the main antitumor drug was 5-fluorouracil which was co-delivered with liposomes. All three studies proved that the liposomal statin nano-formulation had a synergistic effect on cancer therapy and increased the response of the tumor cells to conventional anticancer drugs. In 2018, Hongyue Jin *et al.* used a targeted liposomal formulation of simvastatin and paclitaxel and investigated statin's effect on the reversion of epithelial-mesenchymal transition as an important mechanism of drug resistance.^103^ It was revealed that the nano-formulation of simvastatin suppressed integrin-β3 and focal adhesion formation and led to epithelial–mesenchymal transition reversion. Also, the used nano-formulation causd re-polarization of tumor-associated macrophages and promoted M2-to-M1 phenotype switching. In another targeted liposomal cancer therapy study in 2018, Lucyna Matusewicz and her colleagues used an immunoliposome with simvastatin as a promising therapy in the treatment of breast cancer cells overexpressing HER2.^104^ They attached humanized anti-HER2 antibodies to the surface of the liposome to increase its affinity for HER2-overexpressing breast cancers. The result revealed that targeted liposomal simvastatin not only led to low non-specific cytotoxicity but also had high selectivity towards HER2-overexpressing breast cancer cells.

#### 6.1.2. Micelles

Micelles present several advantages, particularly in enhancing the solubility of hydrophobic drugs, making them highly effective for encapsulating poorly water-soluble compounds and improving their bioavailability. Their small size, typically ranging between 10 and 100 nanometers, allows for superior tumor penetration and accumulation in comparison to larger drug delivery systems such as liposomes. Additionally, micelles can form stable colloidal dispersions, with their capacity for dynamic assembly and disassembly facilitating controlled drug release. Similar to liposomes, micelles can be surface-modified with targeting ligands or PEGylated to extend their circulation time and improve specificity in drug targeting.^105–107^

However, micelles also encounter certain challenges, particularly in terms of their stability *in vivo*. They may become unstable within the bloodstream, and dilution following injection can result in premature drug release, thereby limiting their effectiveness. Another limitation is their relatively low drug-loading capacity, as the hydrophobic core offers limited space for drug encapsulation, potentially reducing their overall therapeutic impact.^106,107^

In the context of cancer therapy, micelles are especially useful for delivering hydrophobic drugs such as paclitaxel and docetaxel, which are otherwise difficult to formulate due to their poor water solubility. Furthermore, micelles have been utilized to combat multidrug resistance in tumors, as their small size and surface characteristics enhance cellular uptake and drug accumulation, leading to improved therapeutic outcomes. Micelles are also employed in combination therapies, co-encapsulating various drugs, such as chemotherapeutics and signaling inhibitors, to achieve synergistic effects in cancer treatment.^108–110^

In 2020, Sandip A. Bandgar *et al.* used micellar simvastatin combined with alendronate sodium as a cytotoxic, cell cycle arresting, and proapoptotic agent.^111^ After achieving the optimized formulation and physically mixing with hydrophilic alendronate sodium, the cytotoxicity, cell cycle arrest, and apoptotic activities were measured. The results revealed that treatment with micellar simvastatin combined with alendronate sodium could significantly inhibit cell growth with low IC50 values against all cells and could inhibit cell multiplication in the S phase. Anbu Mozhi *et al.* investigated the penetration of a proapoptotic and antiangiogenic micellar nanoprobe in a 3D multicellular spheroid for chemotherapy.^112^ After light irradiation, simvastatin and photosensitizer protoporphyrin IX were released to the spheroid and triggered the generation of reactive oxygen species (ROS). According to the results, synergistic apoptosis and necrosis occurred along with the cell cycle arrest. Michael M. Lübtow *et al.* in 2020 studied the blood–brain barrier permeability and cytotoxicity of micellar atorvastatin against Glioblastoma 2D and 3D Models.^113^ There was a significant difference between the concentration of statins in the cerebrospinal fluid (CSF) in free administration atorvastatin and its nanoformulation. Also, the nanoformulation of atorvastatin showed less cytotoxicity against nerve cells.

#### 6.1.3. Nano-emulsions

Nano-emulsions present several advantages, particularly in enhancing the solubility of hydrophobic drugs. As oil-in-water or water-in-oil systems, they are effective in encapsulating poorly water-soluble drugs, thereby improving both solubility and bioavailability. Furthermore, nano-emulsions exhibit long-term stability due to their superior thermodynamic properties compared to other nanocarriers, allowing for prolonged storage. Additionally, their relatively simple formulation and production processes make them highly scalable and cost-efficient for drug delivery applications. The dispersed oil phase within nano-emulsions also permits a higher drug-loading capacity compared to liposomes or micelles, increasing their overall utility.^114,115^

Despite these advantages, nano-emulsions have certain drawbacks. They are rapidly cleared by the reticuloendothelial system, which limits their circulation time within the body. Moreover, their targeting capabilities are more restricted in comparison to liposomes or micelles, as nano-emulsions provide fewer opportunities for functionalization with targeting ligands. There is also a potential risk of toxicity from the emulsifying agents or surfactants used to stabilize nano-emulsions, particularly with repeated administration.^115^

In cancer treatment, nano-emulsions are especially suitable for topical and localized therapies, such as those used in skin cancer or intratumoral injections. They are also highly effective in delivering lipophilic anticancer drugs like curcumin and paclitaxel. Additionally, nano-emulsions hold promise in photodynamic therapy, given their ability to incorporate photosensitizers for targeted cancer destruction.^116,117^

In 2022, Laxman Subedi and his colleagues investigated the enhancement of the anticancer effect of atorvastatin-loaded nanoemulsions by improving oral absorption.^84^ Oral absorption is improved with the aid of multivalent intestinal transporter-targeting lipids. These lipids, such as the ionic complex of deoxycholic acid, cationic lipid 1,2-dioleyl-3-trimethylammonium propane, biotin-conjugated lipid, and d-alpha-tocopherol polyethylene glycol succinate, acted as anchors and led to permeation atorvastatin through the multivalent and bile acid transporters without P-glycoprotein (P-gp)-mediated efflux. With the mentioned formulation, the oral bioavailability increased in comparison to the free oral administration of atorvastatin. The tumor growth was suppressed significantly compared to the control group in 4T1 cell-bearing mice. Also in 2022, Waleed Y. Rizg *et al.* repurposed lovastatin as a cytotoxic agent against the tongue carcinoma HSC3 cell line with a new administration approach.^118^ The administered nanoemulsion included a combination of lovastatin and a eucalyptus oil-based nanoemulgel carrier. The optimized lovastatin-loaded self-nanoemulsifying drug delivery system was loaded into the gelling agent Carbopol 934. The diameter of 85 nm and 93% stability were the characteristics of the optimized nanoemulsion formulation. After measurements and comparisons were made between all formulations of nanoemulsions, the optimized formulation showed the best inhibitory concentration (IC50) and caspase-3 enzyme values. Also, the study revealed the synergistic effect between lovastatin and eucalyptus oil in the treatment of tongue cancer. Mayson H. Alkhatib *et al.* in 2021, to reduce the toxic effects of the anticancer drugs doxorubicin and pravastatin on liver and heart cells, utilized drug delivery *via* nanoemulsions.^119^ The body weight changes and biochemical and histopathological profiles of hepatic and cardiac tissues were monitored. According to the analyses of the recorded measurements, hepatotoxicity and cardiotoxicity reduced significantly compared to free administration of the drugs. In 2019, Huda Mohammed Alkreathy *et al.* investigated the anti-tumor activity of doxorubicin and simvastatin co-delivered in a nanoemulsion formula against Ehrlich ascites carcinoma.^120^ Overall, the results showed that the nanoemulsion formula of doxorubicin and simvastatin induced fewer side effects and acted more efficiently than the free form of the drugs. Almost the same, in another study in 2018, Mayson H. Alkhatib and his colleague investigated the cytotoxicity effect of the nanoemulsion formulation of doxorubicin and pravastatin on MCF-7 Breast Cancer Cells and HFS Human Foreskin Cells.^73^ The results showed that the nanoemulsion formula of doxorubicin and pravastatin induced fewer side effects and acted more efficiently than the free form of the drugs.

#### 6.1.4. Others

Lipid nanoparticles (LNPs), nanostructured lipid carriers (NLCs), solid lipid nanoparticles (SLNs), and all lipid-based nanoparticles in combination with new technologies or other nanoparticles are also included in the category of lipid-based nanoparticles.

Lipid-based nanoparticles present several advantages in drug delivery, primarily due to their biodegradable and non-toxic composition, as they are constructed from physiological lipids that reduce systemic toxicity and promote safe administration. These nanoparticles enable controlled drug release, making them particularly suitable for long-term cancer treatment, as they provide sustained therapeutic effects. In addition, they exhibit high physical stability, minimizing the risk of drug leakage during both storage and circulation, which represents an improvement over liposomal systems. Their size and surface properties also contribute to enhanced tumor accumulation through the enhanced permeability and retention (EPR) effect. Furthermore, lipid-based nanoparticles protect sensitive molecules, such as siRNA or DNA, from enzymatic degradation, which is essential for the effective delivery of genetic therapies.^121,122^

However, lipid-based nanoparticles also have limitations. Their capacity to load hydrophilic drugs is restricted, as they are more efficient in encapsulating hydrophobic compounds. The formulation process can be technically challenging, requiring meticulous control over parameters such as particle size and lipid composition to ensure optimal efficacy. Additionally, the surfactants used to stabilize these nanoparticles may cause irritation or toxicity at high concentrations, posing a potential limitation to their clinical application.^122^

In cancer therapy, lipid-based nanoparticles are highly effective for delivering RNA-based therapeutics, including siRNA and mRNA, which play a critical role in targeting oncogenes and modulating immune responses. These nanoparticles are also advantageous for targeted chemotherapy, as they can be functionalized to achieve active targeting of tumor cells, thereby increasing the precision of drug delivery. Moreover, lipid-based nanoparticles are well-suited for combination therapies, enabling the co-delivery of drugs and genetic materials, which offers a multi-modal approach to cancer treatment.^123–125^

Rita R. Lala and her colleague, in 2021, developed and optimized a solid nanoparticle made of glycerol monostearte (GMS), including atorvastatin calcium and vinpocetine, and evaluated their *in vitro* effect in cancer therapy.^126^ For optimization of the nanoparticle formula, a central composite design was selected. The characteristics of the produced nanoparticles were examined, and their anticancer effect on HepG2, MCF 7, and melanoma B16 F10 cell lines was compared with conventional treatment. This study revealed that the optimized nanoparticle formulation has an enhanced anticancer effect compared with free drugs. In 2018, Vaishali M. Gambhire *et al.* studied the anticancer effect of atorvastatin-loaded lipid nanoparticles on MCF-7 breast cancer cells.^127^ The lipid nanoparticle contains GMS, stearic acid, beeswax, carnauba wax, *etc.* This study showed that the use of lipid nanoparticles for delivery increased the bioavailability of atorvastatin. Also 27.4 μg mL^−1^ and <10 μg mL^−1^ were reported as the total growth inhibition and 50% growth inhibition concentrations respectively.

### 6.2. Polymer-based NPs

#### 6.2.1. Chitosan

Chitosan is a natural polymer derived from chitin, primarily found in the shells of crustaceans. Chitin, the precursor to chitosan, is one of the most abundant biopolymers, and both chitin and its derivative, chitosan, are known for their biocompatibility, biodegradability, and potential in biomedical applications.^128^ These properties make chitosan an ideal candidate for use in nanomedicine. Furthermore, its intrinsic antioxidant, antibacterial, and antitumor properties further enhance its potential for drug delivery, particularly in cancer therapy.^128^ Several studies have explored its anti-cancer properties, showing that it not only inhibits tumor growth but also induces apoptosis in cancer cells.^129^ Chitosan achieves these effects by interfering with cancer cell proliferation and survival, primarily through the activation of various signaling pathways, such as cellular enzymatic mechanisms, antiangiogenic pathways, and the modulation of gene expression. Its cationic nature allows it to interact favorably with the negatively charged cancer cell membranes, which enhances its cellular uptake and provides selective permeability to cancer cells while sparing healthy cells.^129^ One of the major benefits of using chitosan as a carrier for statin-based nanomedicine is its ability to improve drug solubility and stability. Through chemical modifications, such as tailoring its amino, acetamido, and hydroxy groups, chitosan derivatives can exhibit enhanced solubility and anticancer activity.^129^ Chitosan nanoparticles encapsulate statins efficiently, protecting them from degradation and ensuring sustained drug release. Additionally, chitosan can be further chemically modified to enhance its targeting capabilities, enabling precise delivery of statins to cancer cells.^129^ This targeted approach maximizes the therapeutic efficacy of the statin while minimizing off-target effects, thereby reducing potential toxicity to healthy tissues. Overall, chitosan-based nanocarriers provide a versatile platform for more effective and safer cancer therapies.^128,129^ In this way, one study was designed to improve the simvastatin (SMV) efficacy against colon cancer (HCT-116 cells) by using chitosan polymers (CHIT) and Eudragit S100 microparticles (ES100) for better targeting and cytotoxicity. The release of simvastatin double-coated microparticles was found to be influenced by both time and pH. Real-time X-ray radiography of iohexol dye confirmed that the double coat was effective in targeting colonic tissues. The authors reported that the formula of SMV-loaded chitosan coated with Eudragit S100 showed improved pro-apoptotic activity and may be a potential treatment option for colon cancer.^130^ Mallesh Kurakula *et al.* developed an efficient *in situ* gel (ISG) formulation containing chitosan-coated simvastatin (SIM) nanoparticles (NPs) to treat tongue carcinoma (HSC-3 cells) with anti-proliferative activity. Poloxamer 188 and chitosan concentrations were optimized by using a face-centered central composite design (FCCCD). A modified nanoprecipitation method was used to dope SIM-NPs with quercetin (QRC). They found that a mix of 0.24% poloxamer 188 and 0.43% chitosan is the best way to prepare SIM-QRC NP-loaded ISG. The ISG formulation resulted in a considerable increase in apoptosis, mediated by caspase-3 and elevated levels of tumor suppressor proteins.^131^ In an experimental study, Rishabha Malviya *et al.* aimed to create nanoparticles of simvastatin using chitosan-tamarind gum polysaccharide polyelectrolyte complex stabilization and evaluate their effectiveness against human breast cancer (MCF-7 cells). The nanoparticles had high entrapment efficiency and small size. Additionally, egg and tomato membranes were used as biological barriers for drug release, and both membranes showed similar drug release patterns. The nanoparticles were subjected to stability studies for 45 days. Over time, the stability studies also demonstrated less variation in particle size and a decrease in entrapment efficiency. The results showed that they were more effective in controlling the growth of human breast cancer cell lines compared to simple simvastatin.^132^ As mentioned, due to the positive effects that chitosan had on tumor growth and pro-apoptotic activity, a series of studies improved the combination by adding other substances. For example, researchers focused on developing a new drug delivery strategy for targeting liver cancer using erythrocytes loaded with pravastatin-chitosan nanogels (PR-CNG-ER). The PR-CNG-ER cells exposed to phosphatidylserine (PS) exhibited stomatocyte morphology, enhanced entrapment efficiency, and increased fragility. The MTT assay was used to investigate the *in vitro* cytotoxicity of PR-CNG loaded into erythrocytes, using HepG2 cells as a model for HCC. The viability of HepG2 cells was reduced by 28% after incubation with PR-CNG-ER for 72 hours compared to unloaded erythrocytes. These factors suggest that they may have anti-proliferative effects in comparison to free pravastatin.^133^ The researchers also developed Simvastatin nanoparticles crosslinked with tripolyphosphate and chondroitin sulfate in another experimental study. With enhanced oral bioavailability, these Simvastatin chitosan nanoparticles were successfully delivered to hepatocellular carcinomas (HCC) *via* asialoglycoprotein receptor (ASGPR) mediated delivery. Nanoparticles optimized for HepG2 cells demonstrated increased proliferation inhibition and greater cellular uptake *via* ASGPR-mediated endocytosis.^134^

#### 6.2.2. Polyethylene glycol (PEG)

PEG (polyethylene glycol) is a widely used synthetic polymer recognized for its excellent biocompatibility and versatility across various industries, including pharmaceuticals.^135^ One of the key advantages of PEG as a carrier in drug delivery systems is its ability to improve the solubility and stability of hydrophobic drugs, which is crucial for enhancing their bioavailability.^136^ In cancer treatment, PEGylation—the process of attaching PEG chains to molecules—has been shown to prolong the circulation time of drugs by reducing their clearance from the bloodstream.^136^ This “stealth” property, achieved through PEG's hydrophilic nature and ability to evade immune detection, allows nanoparticles to remain in circulation longer, increasing accumulation at the tumor site *via* the enhanced permeability and retention (EPR) effect.^137^ When applied to statin-based nanomedicine, PEGylation can significantly improve the pharmacokinetics and biodistribution of the drug. Statins, originally known for their cholesterol-lowering effects, have demonstrated anti-cancer potential through the inhibition of tumor cell proliferation and induction of apoptosis.^18^ By using PEG as a carrier, the systemic delivery of statins can be enhanced, ensuring controlled and sustained drug release.^137^ This not only reduces the need for frequent dosing but also minimizes the side effects that often result from high drug concentrations.^137^ Additionally, PEGylated nanoparticles can be functionalized with targeting ligands, allowing for precise delivery of statins directly to cancer cells. This targeted approach enhances therapeutic efficacy while minimizing off-target effects and reducing potential toxicity to healthy tissues.^136^ Overall, PEG offers several key benefits in statin-based nanomedicine: it enhances drug solubility, prolongs circulation time, reduces immunogenicity, and allows for controlled drug release, making it a powerful tool in developing safer and more effective cancer therapies.^136,137^ In a study, the main objective of the research was to evaluate the effectiveness of oleoyl macrogol-6-glycerides (Labrafil® M 1944 CS) and caprylocaproylmacrogol-8-glycerides (Labrasol®) as PEG/glycerides in nanostructured lipid carriers (NLCs), in comparison to Labrafac lipophile® WL 1349 which is a PEG-free glyceride. In PBS pH 7.4, dialysis bag diffusion was used to study simvastatin release profiles from freshly prepared and lyophilized Labrafac®, Labrafil®, and Labrasol®-based NLCs. MCF-7 breast cancer cells were used to determine the cytotoxicity of NLCs. The NLC containing Labrasol® exhibited the highest antitumor efficacy with an IC50 of 35.2 ± 3 μg mL^−1^, significantly different (*p* < 0.05) from that of free SV after a 24 hours exposure. This was achieved because Labrasol® caused the cell membrane to become more fluid by disrupting P-gp receptors.^138^ Zhuo Yu *et al.* explained a new strategy for treating hepatocellular carcinoma (HCC) using the drug simvastatin, which targets liver sinusoidal endothelial cells (LSECs) to remodel the tumor microenvironment. The study involved identifying liver sinusoidal endothelial cell (LSEC) features in mouse fibrotic HCC models and human HCC patients, testing the effect of simvastatin on LSECs and hepatic stellate cells (HSCs), and utilizing nanotechnology to deliver simvastatin to LSECs. The study evaluated the effect and toxicity of the nano-drug in mouse models of intrahepatic and hemi-splenic inoculated fibrotic HCC. Using nanotechnology, a targeted delivery system was developed to deliver simvastatin to LSEC cells. As a result of the use of PEG, this system helped to reduce capillarization and regress the stromal microenvironment, while also recruiting natural killer T (NKT) cells through CXCL16 to suppress tumor growth.^139^ Due to the hydrophilic nature of PEG alone, the researchers used polycaprolactone (PCL) with PEG to form self-assembled structures. PCL, on the other hand, is hydrophobic and forms micellar nanoparticles in aqueous media. Therefore, the copolymer containing PCL and PEG can form biodegradable polymeric micelles as a statin carrier. Manjili Hamidreza Kheiri *et al.* conducted an experimental study to compare the impact of two different statins – atorvastatin and rosuvastatin – on breast cancer (MCF-7 cells). As a carrier for statins, biodegradable copolymers (PCL–PEG–PCL) were used to prepare biodegradable polymeric micelles. Several studies were conducted to determine the loading, release, kinetic release, and anti-cancer activity of the drugs. As detected in a cell viability assay, statin-loaded nanoparticles were cytotoxic, whereas unloaded nanoparticles did not show significant cytotoxicity.^140^ In another study, Mehdi Dadashpour *et al.* evaluated the use of polycaprolactone–polyethylene glycol (PCL–PEG) as a nano-carrier to enhance simvastatin (SIM) anticancer activity. Adding SIM to PCL–PEG NPs increased cytotoxicity and sub-G1 cell populations dose-dependently.^141^ Simvastatin-loaded PCL/PEG nanofibers (SIM-PCL/PEG NFs) were examined in a similar study by Elham Norouz Dolatabadi *et al.* to observe if they inhibited resistance to 5-fluorouracil (5-FU) in MKN-45 gastric cancer cells. As determined by the MTT assay, SIM-PCL/PEG NFs showed cytotoxicity toward MKN-45 and MKN-45/R cells. Overall, the findings of this study suggested that SIM-PCL/PEG NFs can induce apoptosis and autophagy in 5-Fluorouracil-resistant MKN-45 (MKN-45/R) cells and have the potential to decrease metastasis.^142^

#### 6.2.3. Poly lactic-*co*-glycolic acid (PLGA)

Poly lactic-*co*-glycolic acid (PLGA) is a biodegradable polymer known for its biocompatibility and controlled release capabilities, making it an excellent choice for drug delivery systems.^143,144^ PLGA degrades into non-toxic byproducts, allowing precise control over drug release rates.^144^ For statin-based nanomedicine, PLGA enhances drug solubility and stability through encapsulation, leading to controlled, sustained release and reduced dosing frequency.^145^ Functionalization of PLGA nanoparticles with targeting ligands further improves statin delivery specifically to cancer cells, boosting efficacy and minimizing side effects.^143,144^ PLGA-based nanoparticles have shown promise in improving drug delivery for cancer treatment by targeting specific cancer processes and improving pharmacokinetics.^145^ Overall, PLGA's controlled release and targeting abilities make it a valuable carrier for statin nanodrugs.^143–145^ According to Naqueeb Anzar *et al.*, simvastatin submicron particles may be a promising treatment option for breast cancer. Spray drying was used to prepare simvastatin-loaded PLGA submicron particles in this study. To obtain high drug loading and formulation yield, different parameters of the nanospray drying method were optimized. As a result of the research, spray drying is an effective method for preparing submicron particles of simvastatin with high drug loading and formulation yield. They showed that simvastatin-loaded PLGA submicron particles (SIM SP) resulted in a considerable increase in AUC_0−24_ (area under the plasma concentration–time curve over the last 24 h dosing interval), *T*_1/2_ (terminal half-life) and *C*_max_ (maximum measured plasma concentration), as well as a decrease in the elimination rate constant (*K*_el_) compared to pure simvastatin.^145^ In addition, there was a study that discussed a nano-based combinational therapy for treating solid tumors, particularly pancreatic cancer. In this study, gemcitabine HCl (GM) and simvastatin (SV) were combined in dual-encapsulated nanoparticles. *In vitro* release studies for 60 hours showed a Higuchi release pattern. Using the MTT assay, the nanoparticles showed lower IC50 than pure drugs in cell toxicity studies. For the pharmacokinetic analysis, a non-compartmental method was used. The bioavailability of gemcitabine and simvastatin in PLGA nanoparticles was improved by 1.4 and 1.3 times, respectively, compared to the drug solution. This indicated that the nanoparticles were successful in delivering the drugs to the intended site, which could potentially enhance their antiproliferative effects. In addition, due to a reduction in the elimination rate constant (*K*_el_) and a longer *T*_1/2_ (terminal half-life), the novel formulation demonstrated better absorption and systemic residence.^146^ In another study, PLGA/PEI nanoparticles were used to enhance the therapeutic efficacy of simvastatin (SMV) and miR-21i (combination agents) in gastric cancers. As compared to free SMV, SMV + miR-21i had significantly lower cell viability, and normal gastric mucosa cells were significantly less cytotoxic. Cell apoptosis of SMV + miR-21i was significantly higher compared to that of individual drugs or miRNA. It is concluded that the combination of SMV and miR-21i (gene therapy and drug therapy) may provide a valuable strategy for the clinical management of gastric cancer.^147^

#### 6.2.4. Hybrid nanoparticles

Hybrid nanoparticles combine organic and inorganic materials to enhance drug delivery and targeting in cancer therapy.^148,149^ In the context of statin-based nanomedicine, hybrid nanoparticles offer several advantages. They allow for controlled and sustained release of statins, improving therapeutic efficacy while reducing dosing frequency. The ability to functionalize these nanoparticles with targeting ligands enables precise delivery of statins to cancer cells, increasing treatment efficiency and minimizing off-target effects.^149,150^ In addition to drug delivery, hybrid nanoparticles can serve dual functions as theranostic agents, integrating both therapeutic and imaging capabilities. This allows for simultaneous cancer treatment and monitoring, making them particularly valuable in precision oncology.^148,149^ Preclinical studies have shown promising results, with hybrid nanoparticles improving drug distribution and tumor targeting, paving the way for more effective cancer therapies.^150^ A hybrid drug delivery system is described in the paper as a possible treatment for prostate cancer (PC-3 cells). As part of the process, SMV is chemically conjugated with acid-terminated poly (d,l-lactic-*co*-glycolic acid) chains before it is converted into nanoparticles (NPs), with more SMV and superparamagnetic iron oxide nanoparticles (SPIONS) incorporated into the PLGA NPs *in situ*. The apoptosis and necrosis mechanisms were assessed in PC-3 cells. SMV anticancer activity against human prostate cancer was significantly enhanced by the PLGA-based hybrid nanocarrier system. By incorporating SPIONs into the nanocarrier, the drug could be guided to tumor cells without causing side effects in other organs.^151^ In addition, Antonella Barone *et al.* conducted an experimental study to create a topical adhesive film that could help treat melanoma. They used simvastatin (SV) as the drug for this purpose. Researchers developed the film using a factorial design approach, which used nanostructured lipid carriers (Ch-NLCs) coated with chitosan. As critical quality attributes, release, permeation, and adhesion were assessed for the SV-Ch-NLC films. *In vitro* cytotoxicity and cutaneous tolerability studies were conducted to ensure film safety and drug effectiveness. The findings revealed that Ch-NLC-incorporated SV in films exhibited enhanced cytotoxic properties, implying that nanostructured films were more effective in inhibiting cell growth than the reference film formula. The Ch-NLC films were found to enhance skin permeation and uptake into melanoma cells, which was attributed to the presence of squalene in the nanoparticle matrix, further supporting the antiproliferative effects of the nanostructured films.^152^

## 7. Challenges and opportunities

**Fig. 4:**
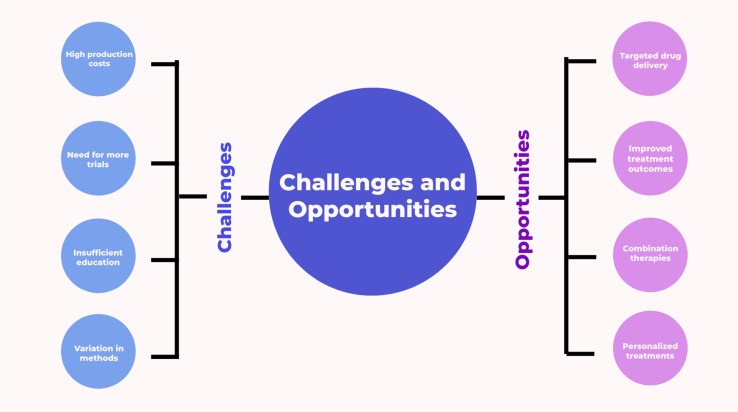
A summary of the challenges and opportunities.

Statin nano-formulations present several challenges that hinder their widespread adoption in clinical practice. One significant challenge is the variation in research methods used in studies, making it difficult to compare results and draw definitive conclusions. Moreover, the complexity involved in the production and formulation of statin nano-formulations demands advanced technologies for processes such as particle size regulation, drug encapsulation, and long-term stability. These technical requirements significantly increase production costs, creating barriers to large-scale implementation. While early studies show promise, more extensive clinical trials are needed to establish the effectiveness and safety of statin-based nano-formulations for real patients. Furthermore, although some preclinical research has shed light on the mechanisms of these nanoparticles, a comprehensive understanding of their biological interactions, particularly in human cancer cells, is still lacking. This uncertainty hinders their acceptance in clinical settings and underscores the need for further investigation. Additionally, the slow adoption of these new nanotechnology treatments is partly due to insufficient education among clinicians. As nanotechnology is still relatively new, many healthcare providers are unaware of its full potential, which contributes to its cautious use in cancer treatment. The conservative nature of medical practices, especially in cancer care, further complicates the integration of these novel treatments into routine clinical use. However, there are exciting opportunities in this field. The exploration of nanoparticles presents new prospects for developing more accurate and personalized drug delivery systems. Nanoparticle formulations of statins could specifically target cancer cells, potentially leading to improved treatment outcomes and reduced side effects. Combining these formulations with other treatments such as immunotherapy or radiotherapy may offer more comprehensive and potent cancer-fighting strategies. A summary of the points mentioned in the text is illustrated in Fig. 4.

## 8. Conclusion

Cancer remains one of the most formidable challenges in modern medicine, characterized by the uncontrolled growth and spread of abnormal cells. Despite significant advancements in cancer treatment, current therapies exhibit limitations that underscore the complexity of the disease. Traditional treatments such as chemotherapy, radiation, and surgery, while sometimes effective in targeting cancer cells, can also harm healthy tissues and cause severe side effects. Moreover, the heterogeneity of tumors and the ability of cancer cells to evolve and develop resistance pose significant barriers. The lack of specificity in some treatments results in collateral damage to normal cells, leading to adverse effects on the patient's overall well-being. Addressing these limitations necessitates continuous research into more precise and personalized approaches to cancer treatment, exploring innovative strategies that can enhance efficacy while minimizing the impact on healthy tissues.

Statins, known as a class of drugs for hypercholesteremia, are promising drugs in cancer treatment according to their special anti-tumor effects such as inhibition of tumor cell growth, inducing apoptosis, inhibition of angiogenesis, inhibition of tumor invasion and metastasis, and reversion of multidrug resistance. Despite being useful in various conditions, statins have some limitations like other drugs. New developments in the field of pharmacology and the convergence between pharmacology and other fields lead to change limitations to opportunities.

On the other hand, nanotechnologies' application in cancer therapy is one of the newest and most high-tech methods used to treat cancers in recent years. The convergence of nanotechnology and pharmacology started a revolution in the treatment of diseases especially in cancer treatment. This convergence starts a new era of investigation about the new effect of nano-drugs on patients and the mechanisms in their bodies. Nanotechnology can help in better drug delivery and can enhance the effect of drugs on their target while at the same time, it can diminish the drugs' side effects. One of the key aspects of our work has been writing this review to highlight the use of nanoparticles in statin delivery and to explore various dimensions of this approach. This review also emphasizes recent progress in statin-loaded nanoformulations, shedding light on their mechanisms of action and how they can be effectively applied in cancer therapy.

Although statins are drugs with high cost-effectiveness and few limitations, using statins in combination with nanotechnology will increase efficacy and reduce side effects. Numerous investigations have been conducted on the utilization of statin nano-formulations in the context of cancer treatment, addressing diverse facets of this scientific domain. These studies have systematically assessed distinct statins in conjunction with a spectrum of nano-formulations, encompassing nanoemulsions, liposomes, micelles, PLGAs, PLAs, PEGs, *etc.* This review endeavors to comprehensively survey and analyze recent noteworthy contributions in the realm of statin nano-formulation applications for cancer treatment.

## 9. Future outlook and prospects

Looking ahead, the integration of nanotechnology with statin therapy offers promising opportunities for more personalized and effective cancer treatments. Future studies should focus on conducting clinical trials to validate the efficacy of these nanoformulations, optimizing dosage, and minimizing long-term side effects. Additionally, exploring the potential of combining statin-loaded nanoparticles with other therapies, such as immunotherapy, could enhance treatment outcomes. This review aims to provide a consolidated reference for researchers, offering key insights to inform and guide future investigations in this evolving field.

## Conflicts of interest

There are no conflicts to declare.

## Data Availability

No primary research results, software or code have been included and no new data were generated or analysed as part of this review.
